# Comparative analysis reveals distinctive genomic features of Taiwan hot-spring cyanobacterium *Thermosynechococcus* sp. TA-1

**DOI:** 10.3389/fmicb.2022.932840

**Published:** 2022-08-11

**Authors:** Yen-I Cheng, Yu-Chen Lin, Jyh-Yih Leu, Chih-Horng Kuo, Hsiu-An Chu

**Affiliations:** ^1^Institute of Plant and Microbial Biology, Academia Sinica, Taipei, Taiwan; ^2^Department of Life Science, Fu Jen Catholic University, New Taipei City, Taiwan

**Keywords:** thermophilic cyanobacterium, *Thermosynechococcus*, genome, comparative genomics, hot-spring cyanobacteria

## Abstract

*Thermosynechococcus* is a genus of thermophilic unicellular cyanobacteria that dominates microbial mats in Asian non-acidic hot springs. These cyanobacteria are the major primary producers in their ecological niches and are promising sources of thermostable enzymes for biotechnology applications. To improve our understanding of these organisms, we conducted whole-genome sequencing of a novel strain for comparative analysis with other representatives in the same genus. This newly characterized strain, *Thermosynechococcus* sp. TA-1, was isolated from the Taian hot springs in Taiwan. Analyses based on average nucleotide identity (ANI) and genome-scale phylogeny suggested that TA-1 and another Taiwanese strain CL-1 belong to a novel species-level taxon. Two metagenome-assembled genomes (MAGs) originated from India represent the sister group, and *Thermosynechococcus elongatus* PKUAC-SCTE542 from China is the next closest lineage. All cultivated strains and MAGs from Japan form a separate monophyletic clade and could be classified into two species-level taxa. Intriguingly, although TA-1 and CL-1 share 97.0% ANI, the genome alignment identified at least 16 synteny breakpoints that are mostly associated with transposase genes, which illustrates the dynamic nature of their chromosomal evolution. Gene content comparisons identified multiple features distinct at species- or strain-level among these *Thermosynechococcus* representatives. Examples include genes involved in bicarbonate transportation, nitric oxide protection, urea utilization, kanamycin resistance, restriction-modification system, and chemotaxis. Moreover, we observed the insertion of type II inteins in multiple genes of the two Taiwanese strains and inferred putative horizontal transfer of an asparagine synthase gene (*asnB*) associated with exopolysaccharides gene cluster. Taken together, while previous work suggested that strains in this genus share a highly conserved genomic core and no clear genetic differentiation could be linked to environmental factors, we found that the overall pattern of gene content divergence is largely congruent with core genome phylogeny. However, it is difficult to distinguish between the roles of phylogenetic relatedness and geographic proximity in shaping the genetic differentiation. In conclusion, knowledge of the genomic differentiation among these strains provides valuable resources for future functional characterization.

## Introduction

Thermophilic cyanobacteria grow photosynthetically and are often the primary producers in non-acidic hot springs (Ward et al., 2012). The underlying mechanisms of how thermophilic cyanobacteria adapt to diverse ecological niches and various stressful conditions of hot springs are still not fully understood (Chen et al., 2021; Prondzinsky et al., 2021). The information would be valuable for developing feasible solutions to mitigate the impacts of global warming (Matos et al., 2021). Additionally, the thermostable enzymes and bioproducts of these bacteria have been utilized in numerous scientific research and biotechnology applications (Leu et al., 2013; Su et al., 2013; Liang et al., 2019; Patel et al., 2019; Zuliani et al., 2021), which further illustrates the importance of these organisms.

Among thermophilic cyanobacteria, the genus *Thermosynechococcus* dominates the microbial mats in Asian non-acidic hot springs in the temperature range of about 50–65°C (Liao et al., 2006; Everroad et al., 2012; Tang et al., 2018; Patel et al., 2019; Prondzinsky et al., 2021). The genome sequences of several representative *Thermosynechococcus* strains have been determined and analyzed. These include *Thermosynechococcus elongatus* BP-1, *Thermosynechococcus vulcanus* NIES-2134, and *Thermosynechococcus* sp. NK55 from Japan (Nakamura et al., 2002; Stolyar et al., 2014); *Thermosynechococcus elongatus* PKUAC-SCTE542 from China (Liang et al., 2019); and *Thermosynechococcus* sp. CL-1 from Taiwan (Hsueh et al., 2007; Cheng et al., 2020; Table 1). The closest known relative of this genus is the *Synechococcus* phylogenetic lineage C1 (Papke et al., 2003), represented by *Synechococcus lividus* PCC 6715, which was isolated from a hot spring in Yellowstone National Park in the United States (Dyer and Gafford, 1961).

**TABLE 1:** List of genome sequences included in the comparative analysis.

Strain	Abbr.	Geographic origin	GenBank accession	Annotation date	Genome size (N50 of draft assembly) (bp)	G + C content (%)	Coding density (%)	No. of protein-coding genes	No. of pseudogenes	No. of rRNA Genes	No. of tRNA Genes	References
*T.* sp. TA-1	TA-1	Miaoli, Taiwan	NZ_CP070960.1	05-MAR-2021	2,658,717	53.6	90.4	2,523	33	3	42	This study
*T.* sp. CL-1	CL-1	Taitung, Taiwan	NZ_CP040671.1	25-SEP-2020	2,647,823	53.5	90.3	2,514	37	3	41	Cheng et al., 2020
*T. elongatus* PKUAC-SCTE542	E542	Sichuan, China	NZ_CP032152.1	03-AUG-2020	2,650,294	53.3	91.0	2,508	35	3	41	Liang et al., 2019
*T. elongatus* BP-1	BP-1	Beppu, Japan	NC_004113.1	13-DEC-2020	2,593,857	53.9	88.4	2,446	35	3	40	Nakamura et al., 2002
*T. vulcanus* NIES-2134	2134	Wakayama, Japan	NZ_AP018202.1	14-DEC-2020	2,571,271	53.9	88.9	2,422	36	3	40	NA
*T.* sp. NK55	NK55	Nagano, Japan	NC_023033.1	17-JAN-2021	2,520,064	53.8	87.1	2,333	78	3	41	Stolyar et al., 2014
J003 k99_1008539	J003	Tokyo, Japan	GCA_003696925.1	29-OCT-2018	2,311,962 (17,448)	53.8	85.0	2,182	148	3	35	Ward et al., 2019
M3746_W2019_013	M3746	Tokyo, Japan	GCA_015296045.1	09-NOV-2020	2,387,168 (53,495)	53.3	89.7	2,258	34	3	39	Alcorta et al., 2020
M46_R2017_013	M46	Ladakh, India	GCA_015296025.1	09-NOV-2020	2,396,365 (16,361)	52.3	72.3	2,155	324	3	41	Alcorta et al., 2020
M55_K2018_012	M55	Chhattisgarh, India	GCA_015295915.1	09-NOV-2020	2,399,209 (27,694)	53.6	90.3	2,343	36	2	39	Alcorta et al., 2020
M98_K2018_005	M98	Chhattisgarh, India	GCA_015295825.1	09-NOV-2020	2,370,950 (35,735)	53.7	90.0	2,290	43	3	39	Alcorta et al., 2020
*S. lividus* PCC 6715	*S. l.*	Yellowstone National Park, United States	NZ_CP018092.1	20-DEC-2020	2,659,739	53.5	80.1	2,383	196	3	41	Tang et al., 2022

The sampling included all cultivated strains in the genus *Thermosynechococcus* with complete genome sequences available. Additionally, metagenome-assembled genomes assigned to this genus were included; these assemblies are identified by only the strain id but not the scientific name. *Synechococcus lividus* PCC 6715 was included as an outgroup. All strains were collected from hot springs.

*Thermosynechococcus* genomes typically have smaller sizes and higher GC content than most mesophilic cyanobacteria (Larsson et al., 2011; Alcorta et al., 2020; Prondzinsky et al., 2021). Recently, a study found that *Thermosynechococcus* strains have a highly conserved genome core, and no clear genetic differences or adaptive mechanisms could be detected when comparing strains isolated from hot springs with different environmental factors (Prondzinsky et al., 2021). This observation contrasts with a study of *Synechococcus* phylogenetic lineage A/B strains from a hot spring in Yellowstone National Park, which identified genetic differentiation between high- and low-light adapted strains (Becraft et al., 2015; Olsen et al., 2015). This difference between these two distinct genera remains to be investigated.

For genomic characterization of *Thermosynechococcus*, we previously studied a strain CL-1 isolated from the Chin-Lun hot spring in Eastern Taiwan (Cheng et al., 2020). CL-1 was genetically divergent from other characterized strains in the same genus and likely represents a novel species-level taxon based on genome-based phylogeny and average nucleotide identity (ANI) analysis. Furthermore, as compared with other strains, CL-1 has distinct genes related to photosynthesis, transporters, signal transduction, the chaperone/usher system, nitric oxide protection, antibiotic resistance, prokaryotic immunity systems, and other physiological processes (Cheng et al., 2020). However, with CL-1 as the only representative of this novel species-level taxon, whether those genetic features are specific at the level of species or strains is unknown. To address this question, we studied another Taiwanese strain, TA-1, which was isolated from the Taian hot springs in Central Taiwan (Leu et al., 2013). TA-1 is able to grow photosynthetically under high levels of CO_2_ (up to 80%) and has been applied to produce C-phycocyanin with thermo- and pH-stability for bio-industrial applications (Leu et al., 2013).

In this work, we determined the complete genome sequence of TA-1 and conducted a comparative analysis with other *Thermosynechococcus* strains to gain a better understanding of these cyanobacteria. In addition to providing a first look into the within-species level diversity of this species-level taxon in Taiwan, we improved upon our previous work (Cheng et al., 2020) by incorporating multiple metagenome-assembled genomes (MAGs) with diverse geographic origins to achieve a better sampling of *Thermosynechococcus* diversity (Table 1). Finally, the updated comparative analysis used newer versions of annotation of these *Thermosynechococcus* genomes. This update is important because the annotation of PKUAC-SCTE542 had a major update that changed the number of intact coding sequences from 1,625 to 2,508, which would greatly impact the inference of the core genome composition of *Thermosynechococcus* (Prondzinsky et al., 2021).

## Materials and methods

### Biological material and growth conditions

The strain TA-1 was isolated from the Tai’an hot springs (40–63°C, pH 7.5) in Miaoli County, Taiwan (Leu et al., 2013). The water sample had a conductivity of 1.97 mS/cm and the concentration (unit: mEq/L) of major ions were: F^–^, 2.69; K^+^, 1.21; Ca^2+^, 0.99; Cl^–^, 26.96; Mg^2+^, 0.96; Na^+^, 478.12; Si^4+^, 11.83; SO_4_^2–^, 5.47; HCO_3_^2–^, 1044.43 (Central Geological Survey, Ministry of Economic Affairs, Taiwan). The strain underwent axenic culture at 50°C in BG-11 medium supplemented with 20 mM TES (pH 8.0) under continuous white LED light (20 μmol photons m^–2^ s^–1^). For antibiotic resistance experiments, the *Thermosynechococcus* cultures were grown on BG-11 solid agar plates (5 mM TES, pH 8.0) containing 5 μg/ml of kanamycin. For phototaxis experiments, the TA-1 and CL-1 cultures were grown on BG-11 solid agar plates (5 mM TES, pH 8.0) under directional white light.

### Microscopy

The procedures for microscopy were modified from those described in our previous work (Cho et al., 2019). Briefly, for scanning electron microscopy, the samples were fixed in 2.5% glutaraldehyde and 4% paraformaldehyde in 0.1 M sodium phosphate buffer, pH 7.0 at room temperature for 1 h. After rinsing with buffer three times for 20 min, samples were dehydrated in an ethanol series. Critical point drying was done with a Leica EM CPD300 critical point dryer. After coating with a Hitachi E-1010 ion sputter, an FEI Quanta 200 scanning electron microscope at 20 kV was used for viewing and imaging.

For transmission electron microscopy, cultured cells were frozen using high-pressure freezer (Leica EMPACT2) at 2,000–2,050 bar. Freeze substitution was performed in anhydrous acetone (containing 2% OsO4) using a Leica EM AFS2. The samples were kept at −90°C for 2 days, at −60°C for 12 h, at −20°C for 12 h, 0°C for 12 h, and raised to room temperature. After rinsing with acetone two times (2 h each time), the samples were infiltrated and embedding with the Spurr’s resin. Ultra-thin sections (70–90 nm) were cut using a Reichert UltraCut S or Leica EM UC7 and collected using 100 mesh copper grids. The sections were stained with 5% uranyl acetate in 50% methanol for 10 min and 0.4% lead citrate for 4–6 min. Sections were examined using a Philips CM 100 transmission electron microscope at 80 kV and the images were captured with a Gatan Orius CCD camera.

### Genome sequencing and analysis

The procedures for genome sequencing and analysis were based on those described in our previous work (Cheng et al., 2020; Weng et al., 2021). All bioinformatics tools were used with the default settings unless stated otherwise.

Briefly, total genomic DNA was prepared from 150 ml of liquid culture grown for 5 days using the DNeasy Plant Maxi Kit (QIAGEN, Germany). The quantity and quality of the DNA sample were assessed with a NanoDrop 2000 spectrophotometer (Thermo Fisher, United States) and 1% agarose gel electrophoresis. Two sequencing platforms were used for whole-genome shotgun sequencing, namely, Illumina short reads and Oxford Nanopore Technologies (ONT) long reads. For Illumina sequencing, a paired-end library was prepared by using KAPA LTP Library Preparation Kit (KK8232; Roche, Switzerland) without amplification, then sequenced by using a HiSeq X Ten sequencer. For ONT sequencing, the library was prepared by using the ONT Ligation Kit (SQK-LSK109) and sequenced by using MinION (FLO-MIN106; R9.4 chemistry and MinKNOW Core v3.6.0); Guppy v3.4.5 was used for base calling. The raw reads from both platforms were combined for *de novo* assembly by using Unicycler v0.4.8-beta (Wick et al., 2017). For validation, the Illumina and ONT raw reads were mapped to the assembly by using BWA v0.7.12 (Li and Durbin, 2009) and Minimap2 v2.15 (Li, 2018), respectively. The results were programmatically checked by using SAMtools v1.2 (Li et al., 2009) and manually inspected by using IGV v2.3.57 (Robinson et al., 2011). The finalized assembly was submitted to the National Center for Biotechnology Information (NCBI) and annotated by using their Prokaryotic Genome Annotation Pipeline (PGAP) (Tatusova et al., 2016).

For comparative analysis, a list of *Thermosynechococcus* strains with complete genome sequences available from GenBank (Benson et al., 2018) was compiled (Table 1). Furthermore, multiple MAGs that may be assigned to *Thermosynechococcus* (Alcorta et al., 2020; Prondzinsky et al., 2021) were included to improve the sampling of genetic, phylogenetic, and geographic diversity. One MAG, OHK43 from Japan, was omitted because this assembly (accession JACOMP000000000) has > 99.6% ANI with NK55, is highly fragmented with 273 contigs, and lacks annotation. *S. lividus* PCC 6715, which has been demonstrated as a sister lineage of *Thermosynechococcus* (Liang et al., 2019; Komarek et al., 2020; Tang et al., 2022), was included as an outgroup. The annotation was based on those provided in the GenBank files. The homologous gene clusters (HGCs) among these genomes were identified by using OrthoMCL v1.3 (Li et al., 2003).

For phylogenetic analysis, MUSCLE v3.8.31 (Edgar, 2004) was used to generate multiple sequence alignments and PhyML v3.3 (Guindon and Gascuel, 2003) for maximum likelihood inference. The proportion of invariable sites and the gamma distribution parameter were estimated from the dataset, and the number of substitute rate categories was set to four. To quantify genome similarity, FastANI v1.1 (Jain et al., 2018) was used to calculate the percentage of genomic segments mapped and the ANI based on these segments for each genome pair. For pairwise genome alignment, the NUCleotide MUMmer (NUCmer) program of the MUMmer package v3.23 (Kurtz et al., 2004) was used with the setting “–maxmatch –mincluster 2000.” Transposase genes based on annotation keyword search were used as queries to search the entire genome sequences using TBLASTN v2.11.0 + (Camacho et al., 2009). Only hits with high-scoring pairs accounting for at least 70% of the query length and overall amino acid sequence identity of at least 90% were retained.

The packages ggplot2 v3.3.3 (Wickham, 2009) and genoplotR v0.8.9 (Guy et al., 2010) were used to visualize the organization and synteny conservation of selected genes.

## Results and discussion

### Morphology of *Thermosynechococcus* sp. TA-1 cells

The cell morphology of *Thermosynechococcus* sp. TA-1 is shown in Supplementary Figure 1. Consistent with the previous description (Leu et al., 2013), TA-1 is unicellular and has rod-shaped cells that are 1.2–2.5 μm in width and 6.0–9.0 μm in length.

### Complete genome sequence of *Thermosynechococcus* sp. TA-1

The whole-genome shotgun sequencing of *Thermosynechococcu*s sp. TA-1 generated 2,432,241*2 pair-end Illumina reads and 73,708 ONT long reads (average length = 15,070 bp, N50 length = 24,058 bp, and total length = 1,110,800,338 bp). These reads provided ∼272-fold (Illumina) and ∼401-fold (ONT) coverage for the single 2,658,717-bp circular chromosome produced by *de novo* assembly. No plasmid was found. The annotation includes 42 tRNA genes, one complete set of 16S-23S-5S rRNA genes, and 2,523 protein-coding genes.

### Comparison with other strains

A total of 1,359 HGCs were shared by all of the genome sequences compared (Table 1). When the outgroup and the MAGs were excluded, the six *Thermosynechococcu*s strains with complete genome sequences available shared 2,058 HGCs, which accounted for ∼84% of the gene content in each individual strain. This finding differs substantially from our previous result that only 1,264 HGCs could be identified among *Thermosynechococcu*s representatives (Cheng et al., 2020), mainly because of the early version of PKUAC-SCTE542 genome annotation contains 944 pseudogenes and only 1,625 intact protein-coding genes. With the updated annotation of this genome, our new result is consistent with a recent inference that *Thermosynechococcus* has a highly conserved core genome (Prondzinsky et al., 2021).

**FIGURE 1:**
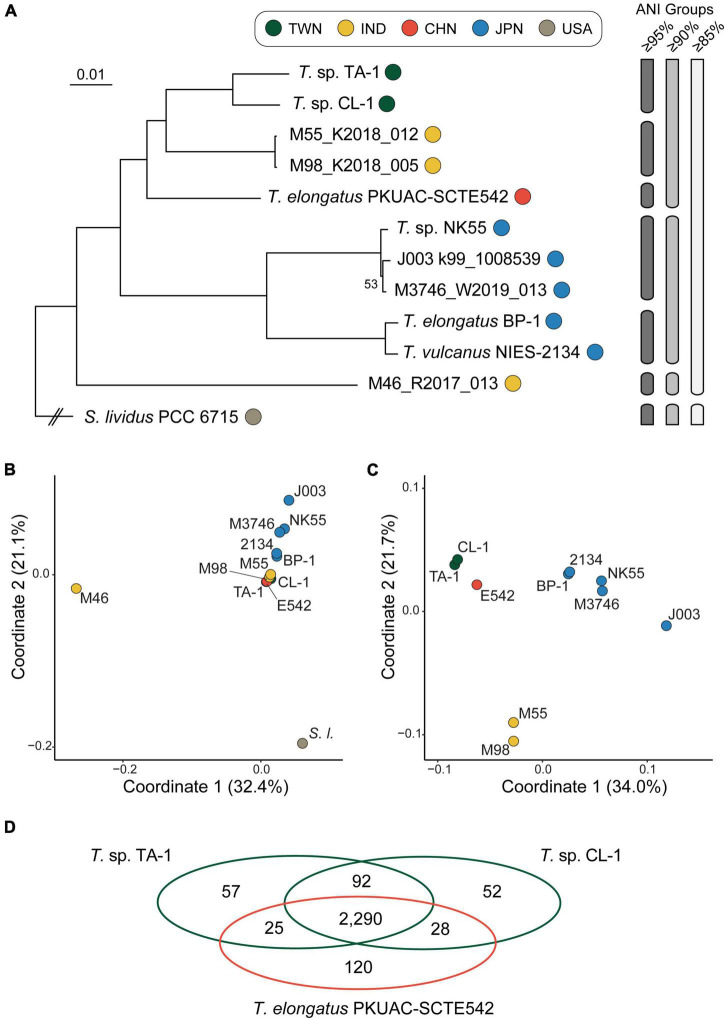
Relationships among representative *Thermosynechococcus* strains. The strains are color-coded according to the geographic origin. TWN, Taiwan; IND, India; CHN, China; JPN, Japan; USA, United States of America. The metagenome-assembled genomes are identified by only the strain names but not the scientific names. **(A)** Maximum-likelihood phylogeny based on 1,311 single-copy genes conserved among all genome sequences analyzed. The concatenated alignment of 383,962 aligned amino acid sites. The bootstrap support was inferred based on 1,000 replicates, the only one internal branch that received < 100% support was labeled. The grouping of strains based on different thresholds of average nucleotide identity (ANI) is illustrated on the right. **(B)** Gene content dissimilarity among all strains based on principal coordinate analysis. **(C)** Gene content dissimilarity, excluding the outgroup and a highly divergent strain M46_R2017_013 to improve the resolution. **(D)** A Venn diagram illustrates the numbers of shared and unique homologous gene clusters among three focal strains.

According to the 1,311 HGCs present as single-copy genes in all strains analyzed, we obtained a concatenated alignment containing 383,962 aligned amino acid sites. A maximum-likelihood phylogeny inferred from this alignment received 100% bootstrap support for all except one branch (Figure 1A). Based on this phylogeny and all possible pairwise ANI analyses, TA-1 was most closely related to another Taiwanese strain, CL-1, and these two strains shared 97.0% ANI, which is above the 95% threshold suggested for delineating bacterial species (Jain et al., 2018). Two MAGs collected in India, M98_K2018_005 and M55_K2018_012, were inferred as the sister species of the Taiwanese lineage.

Among the cultivated strains, PKUAC-SCTE542 from China represented the most closely related species, with ∼90.1% ANI when comparing the two Taiwanese strains. The three cultivated strains from Japan all had smaller genome sizes (Table 1) and belonged to a monophyletic clade that included two species-level taxa based on the 95% ANI threshold (Figure 1A). One MAG collected in India, M46_R2017_013, represented the basal lineage of *Thermosynechococcus*. For the comparison of overall genome sequence similarity, all *Thermosynechococcus* representatives shared > 85% ANI, while *S. lividus* PCC 6715 fell below this threshold. The ANI pattern, as well as the comparison of overall gene content (Figure 1B), both suggested that PCC 6715 is a suitable outgroup. Because M46_R2017_013 and PCC 6715 were both highly divergent from others in the overall gene content, and we conducted a second comparison that excluded these two genomes to improve resolution (Figure 1C). Although those two Indian MAGs represented the sister group to the Taiwanese strains based on the phylogeny of conserved genes, their gene content is quite different. This observation may be due to the nature of incompleteness (and possible contamination) of MAGs and requires future improvement in the availability of complete genome sequences for further investigation.

The taxonomy of these cyanobacteria has been under active revision in recent years (Liang et al., 2019; Komarek et al., 2020; Prondzinsky et al., 2021; Tang et al., 2022). To avoid confusion, we utilized strain names as the major identifiers in this work and described the scientific names based on the associated GenBank records (Table 1) when necessary for trackability. Nevertheless, based on the results of genome-scale phylogeny and overall genome similarity as quantified by ANI, it appeared that further taxonomic revision may be necessary. For example, given the high level of genome similarity (i.e., ANI = 99.0%) between the representative strains of *T. elongatus* and *T. vulcanus*, the relationship between these two species needs to be clarified. Furthermore, with BP-1 as the type strain of *T. elongatus* and its low level of genome similarity (i.e., ANI = 87.7%) with the Chinese strain PKUAC-SCTE542, the species assignment of PKUAC-SCTE542 needs to be re-examined. Finally, based on the comparisons of ANI and gene content (Figure 1), it is reasonable to classify all in-group strains to the same genus (i.e., *Thermosynechococcus*), while excluding *S. lividus* PCC 6715.

Another interesting observation is that there appeared to be some links between the phylogenetic and genomic diversity of these cyanobacteria with geography (Papke et al., 2003). Specifically, all Japanese lineages form a strongly supported monophyletic clade and are relatively similar in overall genome sequence and gene content (Figure 1). However, it is difficult to distinguish between the roles of phylogenetic relatedness and geographic proximity in shaping the genetic differentiation. For future work, more extensive sampling from China and other regions are necessary to better understand the genetic diversity and phylogeography of these cyanobacteria.

For comparison among TA-1 and its two close relatives (i.e., CL-1 and PKUAC-SCTE542), a total of 2,290 HGCs were identified (Figure 1D). Consistent with the patterns observed from molecular phylogeny and ANI analysis, the two Taiwanese strains share more genes with each other than with the Chinese strain. Genes exhibiting strain- or species-specific patterns of presence/absence that may have functional significance are discussed in the following sections. The two Indian MAGs were not used in this comparison due to the concern of genome assembly quality, as well as the limitation of lacking cultured biological materials for follow-up experiments.

**FIGURE 2:**
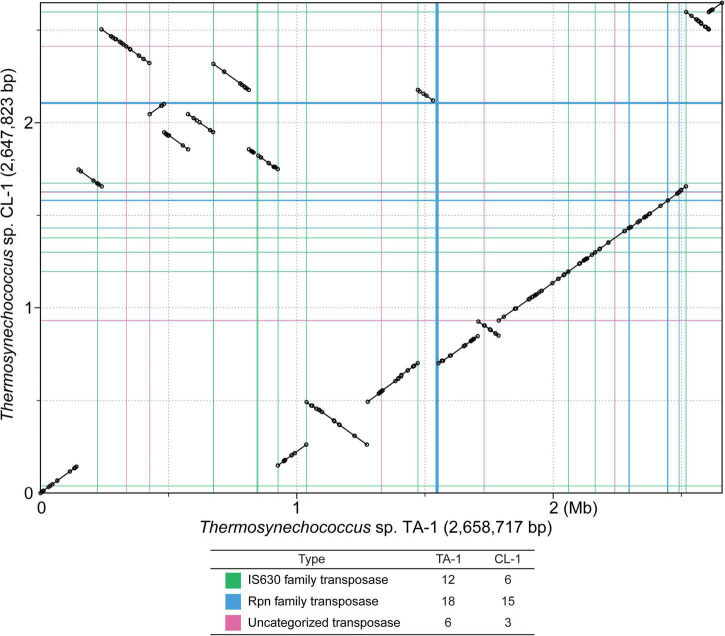
Genome alignment between two *Thermosynechococcus* strains collected in Taiwan. The circular chromosomes are plotted in linear form to illustrate syntenic regions. Positions of putative transposase genes are plotted using colored lines to illustrate the correspondence with synteny breakpoints. Counts of putative transposase genes are provided below the synteny plot. The locations and counts include intact transposase genes listed in the GenBank annotation (Table 1), as well as un-annotated fragments that were identified by TBLASTN searches (see section “materials and methods”).

Intriguingly, despite the close genetic relatedness between the two Taiwanese strains, pairwise genome alignment identified at least 16 synteny breakpoints corresponding to the translocation or inversion of large chromosomal segments (Figure 2). Examination of gene content identified large numbers of putative transposase genes scattered across the entire chromosomes of these strains (i.e., 36 in TA-1 and 24 in CL-1), and nearly all of the synteny breakpoints were associated with transposase genes. The dynamic nature of the chromosomal organization in this genus was reported previously from between-species comparisons (Cheng et al., 2020). The new result from this study further establishes that chromosomal rearrangements are rampant at the within-species level and may be facilitated by mobile genetic elements.

### Overview of notable genetic differentiation

**FIGURE 3:**
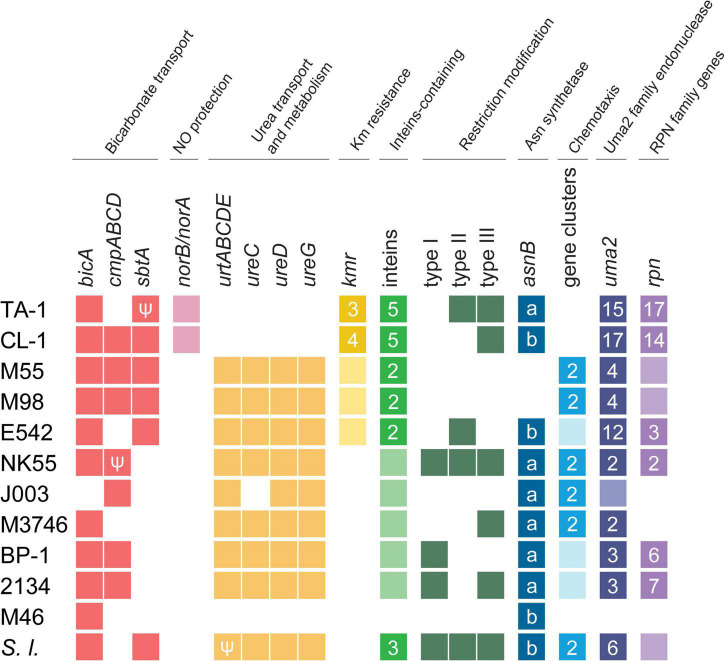
Overview of notable genetic differentiation among representative *Thermosynechococcus* strains. Strain names are abbreviated according to Table 1; *Synechococcus lividus* (*S. l.*) is included as an outgroup. The gene copy numbers are presented in a heat map (absent, white background; single-copy, light-color background; multi-copy, dark-color background with the copy number labeled). Pseudogenes are indicated with the “Ψ” symbol. For the asparagine synthetase gene (*asnB*), the sequence types (i.e., “a” or “b”) are labeled. For *uma2* and *rpn*, the counts include only intact homologs listed in the GenBank annotation (Table 1).

Notable genetic differentiation among these strains are summarized in Figure 3. Consistent with the phylogeny, the two Taiwanese strains TA-1 and CL-1 share several derived traits. For example, these strains appear to have acquired multiple genes involved in nitric oxide protection and kanamycin resistance. In contrast, these strains seem to have lost genes involved in urea transport and chemotaxis. More detailed information regarding the inferred function, distribution patterns, and molecular evolution of these genes are provided in the following sections.

### Bicarbonate transporters

**FIGURE 4:**
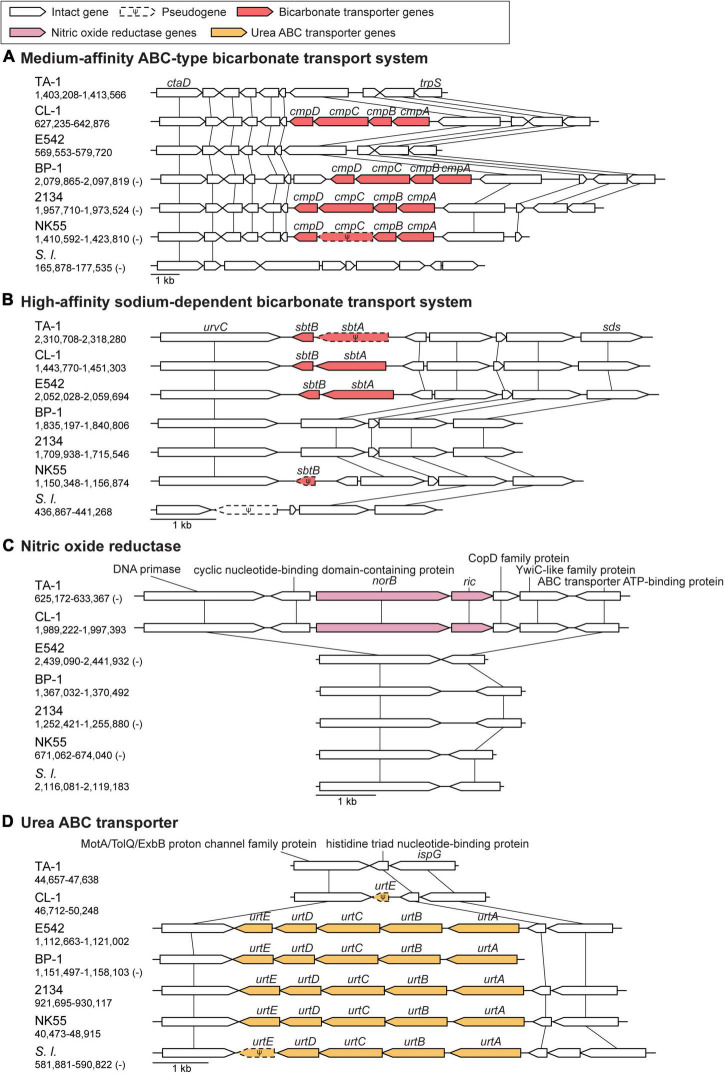
Synteny plots of notable genes. Chromosomal locations are labeled below strain name abbreviations on the left. Homologous genes are linked by vertical lines. **(A)** ABC-type bicarbonate transport system. **(B)** Sodium-dependent bicarbonate transport system. **(C)** Nitric oxide reductase gene cluster. **(D)** Urea ABC transporter gene cluster.

Bicarbonate transporters play an essential role in the CO_2_-concentrating mechanism (CCM) of cyanobacteria to actively import dissolved inorganic carbon (Ci) from aquatic environments for their photosynthetic growth (Price et al., 2008; Gupta et al., 2020). Three types of bicarbonate transporters (BicA, BCT1, and SbtA) have been identified in cyanobacteria (Liu et al., 2021). BicA is a constitutive sodium-dependent, low-affinity, high-flux transporter (Wang et al., 2019), and the corresponding gene *bicA* was conserved in all *Thermosynechococcus* strains analyzed except for one MAG (Figure 3). In comparison, BCT1 (encoded by the *cmpABCD* operon) is an inducible, medium-affinity, low-flux ABC transporter (Omata et al., 1999). The *cmpABCD* operon is present in CL-1 (locus tags FFX45_RS03210-25) and all three Japanese strains but not TA-1, PKUAC-SCTE542, or the outgroup *S. lividus*. According to the syntenic conservation (Figure 4A), this gene cluster may be present in the most recent common ancestor (MRCA) of *Thermosynechococcus*, and there were two independent losses in TA-1 and PKUAC-SCTE542. Finally, SbtA is an inducible sodium-dependent, high-affinity, low-flux bicarbonate transporter (Liu et al., 2021). The *sbtA* gene forms an operon with the neighboring *sbtB* gene. SbtB is a PII-like signaling protein that functions as a Ci sensor. The *sbtA/B* cluster is present in CL-1 (FFX45_RS07275-80), TA-1 (JW907_RS11360-65), PKUAC-SCTE542 (D3A95_RS10120-25), and two Indian MAGs, but absent in all Japanese strains (Figure 3). However, a pseudogenized *sbtB* was found in NK55 (Figure 4B), suggesting that this gene cluster might be present in the ancestor of those Japanese strains.

Of note, the *sbtA* homolog in TA-1 is pseudogenized owing to a 14-bp insertion that resulted in a premature stop codon. TA-1 was originally selected under a high CO_2_ (20% CO_2_) growth condition for high CO_2_ mitigation (Leu et al., 2013). This growth condition may have reduced the need for a high-affinity bicarbonate transporter and relaxed the selection pressure to prevent mutation accumulation in this gene. In comparison, CL-1 was isolated directly from a high-pH and high-temperature environment in the Chin-Lun hot spring (pH 9.3, 62°C) without further selection (Cheng et al., 2020). CL-1 is the only cultivated *Thermosynechococcus* strain known to have all three types of bicarbonate transporters, which suggests that this strain may have a high capacity or flexibility for bicarbonate uptake. Consistent with this expectation, we found that CL-1 had a faster growth rate than TA-1, particularly when supplemented with 100 mM NaHCO_3_ (Supplementary Figure 2).

### Nitric oxide protection

Nitric oxide (NO) is an important signaling biomolecule as well as an intermediate produced in the process of denitrification (Shiro, 2012). NO reductases catalyze the reduction of NO to N_2_O, which function in nitrogen metabolism and a defense against NO toxicity (Büsch et al., 2002; Shiro, 2012). The product of NO reductase, N_2_O, is a powerful green-house gas that contributes to ozone level depletion and global warming (Ravishankara et al., 2009). TA-1 and CL-1 share a distinct gene cluster for NO protection, which is absent in other *Thermosynechococcus* strains (Figure 3). This cluster contains two genes: *norA* (or *dnrN*; JW907_RS02870 in TA-1 and FFX45_RS09820 in CL-1), which encodes an iron-sulfur cluster repair di-iron protein, and *norB* (JW907_RS02875 and FFX45_RS09815), which encodes a quinol type NO reductase. According to the syntenic regions (Figure 4C), these two genes and three other adjacent genes may have been acquired in the MRCA of these two Taiwanese strains. These genes may provide defense against the toxicity of NO molecules released by other microbes in the environments of Taiwan hot springs (Büsch et al., 2002).

### Urea and other nitrogen sources

TA-1 and CL-1 both lack the *urtABCDE* operon that encodes the urea ABC transport system and several urease genes (i.e., *ureA*, *ureD*, and *ureG*) (Veaudor et al., 2019), which are conserved in other *Thermosynechococcus* strains. According to the gene distribution pattern (Figure 3) and the syntenic conservation (Figure 4D), the gene absence is best explained by gene losses in these two Taiwanese strains. Consequently, these two strains are expected to lose the ability to directly use urea as a nitrogen source. However, one previous study reported that TA-1 cells were able to grow slowly in the growth medium supplemented with 17.5 mM urea as the sole nitrogen source (Leu et al., 2013). Because urea is known to decompose slowly into cyanate and ammonia in solution at higher temperature (Hagel et al., 1971; Sun et al., 2014), the derived ammonia may be used by TA-1 cells.

In comparison, the *nir*-*nrtABCD*-*narB* operon (Flores et al., 2005), encodes a nitrite reductase, nitrate ABC transport system, and nitrate reductase, is conserved among all of the *Thermosynechococcus* genomes examined, which suggests that nitrate/nitrite are conserved nitrogen sources among these cyanobacteria. Consistent with this gene content analysis, TA-1 grew equally well in the growth medium supplemented with 5 mM nitrate or nitrite as the nitrogen source (Supplementary Figure 3). Furthermore, all strains have genes that encode ammonium transporters. No nitrogenase gene was identified in any of these genomes, which suggests that they lack the ability for nitrogen fixation.

### Kanamycin resistance

Genes involved in antibiotic production and resistance play important roles in microbial competition and survival in natural environments (Kunhikannan et al., 2021). However, our understanding of the occurrence and evolution of antibiotic resistance genes in thermophilic cyanobacteria that reside in hot-spring environments is still limited (Najar et al., 2020; Sharma et al., 2020). In this work, we found putative bipartite aminoglycoside nucleotidyltransferase gene clusters that may confer kanamycin resistance (Lehmann et al., 2003) in TA-1 (three sets: JW907_RS08580-85, JW907_RS09145-50, and JW907_RS11865-70), CL-1 (four sets; FFX45_RS04515-20, FFX45_RS05120-25, FFX45_RS07775-80, and FFX45_RS11395-400), and PKUAC-SCTE542 (one set: D3A95_RS08325-30) but were absent in all Japanese strains (Figure 3). Each of these clusters contains two genes: one encoding a substrate binding domain and the other nucleotide-binding domains of kanamycin nucleotidyltransferases. Consistent with the expectation derived from gene content, we found that both TA-1 and CL-1 can grow in medium containing 5 μg/ml of kanamycin (Supplementary Figure 4). The gene copy number increases observed in these two Taiwanese strains may suggest a higher selective pressure for antibiotic resistance in their natural environments.

### Intein-containing genes

**FIGURE 5:**
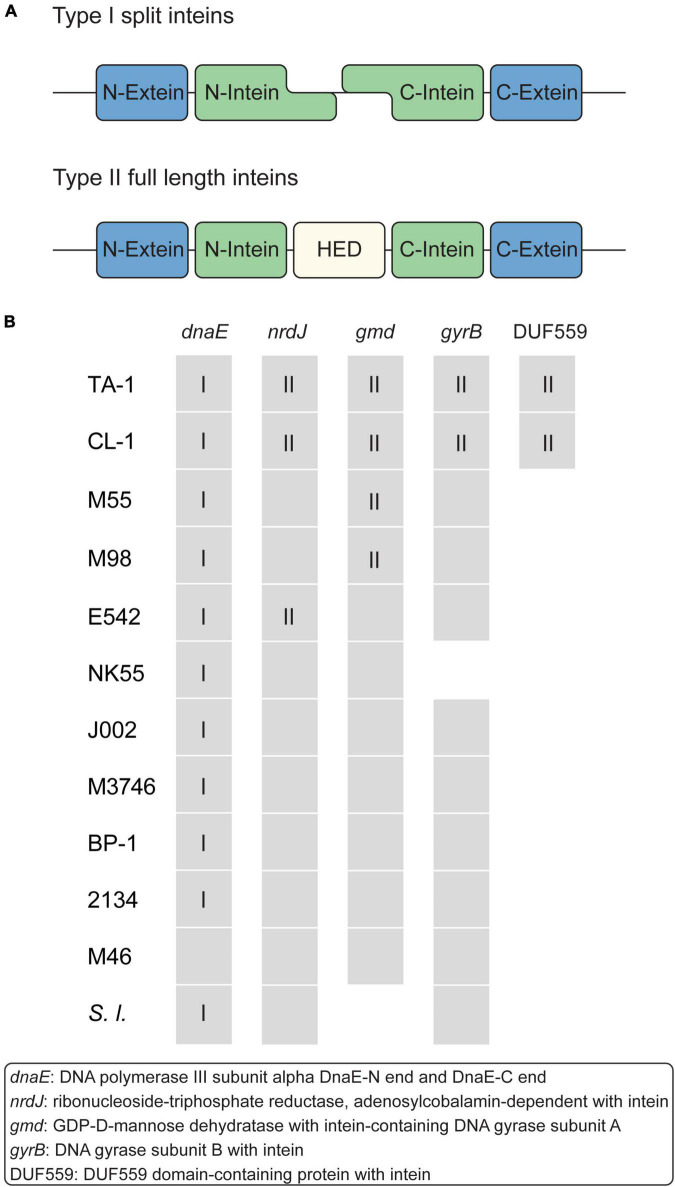
Intein-containing genes. **(A)** Structure of type I and II inteins. **(B)** Distribution of intein-containing genes in strains. Gene presence and absence are indicated as a colored and white background, respectively. For genes that contain inteins, the intein types are indicated by Roman numerals.

Intein is a polypeptide segment that can splice itself spontaneously and precisely from the precursor protein and ligate its flanking polypeptide segments (Baralle and Buratti, 2017). Intein-containing genes are distributed sporadically in all domains of life, but their biological function remains elusive (Nanda et al., 2020). According to the structures, inteins can be classified into three major types (Nanda et al., 2020). Type I split inteins are expressed as two separate polypeptides and undergo splicing in *trans* (Figure 5A). Type II (full length) inteins have a homing endonuclease domain (HED) embedded in between the splicing domains (Figure 5A); the HED may function in the mobilization and propagation of inteins (Nanda et al., 2020). Type III mini-inteins have a contiguous protein splicing domain but lack a HED.

Among these *Thermosynechococcus* genomes, a type I split intein is conserved in the *dnaE* homologs of all strains except for the basal Indian MAG M46_R2017_013 (Figure 5B). This intein-containing *dnaE* is also found in a few other cyanobacteria, namely, *Synechocystis* sp. PCC6803 (Caspi et al., 2003). Notably, multiple insertion events of type II inteins likely occurred in the MRCA of the Taiwanese strains (in *nrdJ* and *gyrB*) and the MRCA of the Taiwanese/Indian strains (in *gmd*, which encodes GDP-D-mannose dehydratase) (Figure 5B). Also, a separate event of type II intein insertion may have occurred to the Chinese strain in *nrdJ*. The DUF559-containing gene belongs to the EDxHD family, which represents a novel homing endonuclease family (Taylor et al., 2011), was identified in only those two Taiwanese strains. The origin and function of these inteins are unclear, and no type III mini-intein was identified in these *Thermosynechococcus* genomes.

### Prokaryotic immunity systems

There are diverse distributions of CRISPR-Cas and restriction-modification systems among these *Thermosynechococcus* strains (Figure 3) that can confer resistance to phages (Makarova et al., 2011; Oliveira et al., 2014). Type I CRISPR-Cas systems were found in TA-1 (JW907_RS04965-04995), CL-1 (FFX45_RS02320-02350), PKUAC-SCTE542 (D3A95_RS09885-09915), and NK55 (NK55_RS04045-04075). Type III-B CRISPR-Cas systems were found in TA-1 (JW907_RS00465-00485), CL-1 (FFX45_RS00470-00490), and PKUAC-SCTE542 (D3A95_RS05650-05670). BP-1 and NIES-2134 do not have any CRISPR-Cas system. Furthermore, TA-1 has one additional gene for CRISPR-associated helicase Cas3′ (JW907_RS09100), which has an HD-like endonuclease domain located in the N-terminal region of Cas3. This gene is absent in all other *Thermosynechococcus* genomes.

For restriction-modification systems (Oliveira et al., 2014), genes that encode types I, II, and III systems have a patchy distribution among the genomes compared (Figure 3). These genes are mostly absent in the MAGs analyzed. However, such gene absence may be artifacts of incomplete assemblies. Among the strains with complete genome sequences available, only the Japanese strain NK55 and the outgroup *S. lividus* PCC 6715 have all three systems. These patterns suggest that multiple gene gain/loss events occurred in the evolutionary history of *Thermosynechococcus.*

### Exopolysaccharide and associated asparagine synthetase genes

**FIGURE 6:**
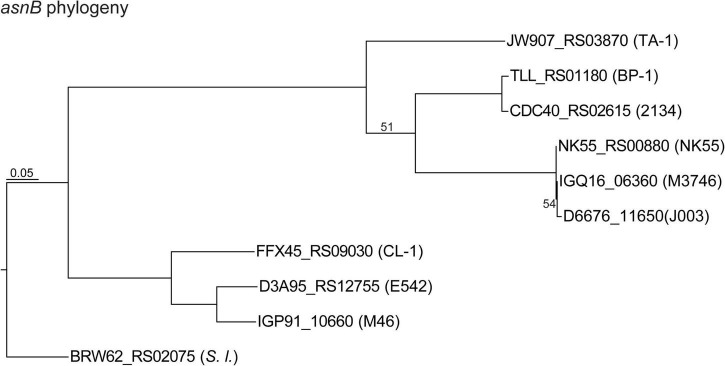
Maximum likelihood phylogeny of the asparagine synthetase gene (*asnB*) homologs. The homologs are labeled with the locus tags; strain name abbreviations are in parentheses.

Exopolysaccharides (EPSs) on the cell surfaces of cyanobacteria may act as barriers to different types of stress and play significant roles in intra- as well as inter-species interactions (Kehr and Dittmann, 2015). Most *Thermosynechococcus* genomes (except for NK55) have one uncharacterized EPS gene cluster, which includes a gene that encodes a glutamine-hydrolyzing asparagine synthase (AsnB; also known as an exosortase). AsnB may be involved in N-linked protein glycosylation of EPS molecules (Bai et al., 2008). The size and composition in the EPS gene cluster vary among these *Thermosynechococcus* strains. Particularly, the components in the EPS gene cluster of TA-1 differ from those in CL-1 and other *Thermosynechococcus* strains. Among the 17 genes in this TA-1 gene cluster, five are uniquely present in this strain and lack identifiable homologs in all other *Thermosynechococcus* strains, suggesting that horizontal gene transfer may be involved in shaping the molecular evolution of this cluster. In addition, the *Thermosynechococcus* AsnB phylogeny (Figure 6) indicated that even for a conserved gene, the homologs among different strains may be classified into two sequence types (Figure 3). Notably, the TA-1 homolog is more similar to those of the Japan strains (71–74% amino acid sequence identity) than the more closely related CL-1 and PKUAC-SCTE542 (about 60% amino acid sequence identity). Although recombination may provide one explanation for this conflict between the gene tree (Figure 6) and species tree (Figure 1A), the exact mechanism is unclear given the apparent geographic isolation and overall genome divergence. Alternatively, the conflict may also be explained by selection or incomplete lineage sorting. It is unclear which of these hypotheses is more plausible.

### Phototaxis and motility

Most *Thermosynechococcus* genomes have three or four sets of gene clusters for motility-related two-component signaling systems (Nakamura et al., 2002; Bhaya, 2004; Ishizuka et al., 2006; Cheng et al., 2020). However, TA-1 and CL-1 contain only the *pixJ-* and *pilJ*-associated two-component signaling systems that are conserved in other *Thermosynechococcus* strains except for NIES-2134. *PixJ* homolog-associated (blue-light-responsive) two-component signaling systems are truncated in NIES-2134 (Ishizuka et al., 2006). The presence of a blue-light-responsive signaling system in cyanobacteria enables them to move toward optimal light conditions and away from UV and excess light conditions (Bhaya, 2004; Conradi et al., 2020). Of note, TA-1 has a truncated *cheA* (*pilL* homolog) in a *pilJ*-associated two-component signaling system (Yoshihara et al., 2002), which may explain its non-motile phenotype (Supplementary Figure 4). Moreover, CL-1 has a truncated *pilC* of the type IV pilus machinery (Bhaya et al., 2000) and is also non-motile (Supplementary Figure 4). Finally, TA-1 and CL-1 both lack the entire gene cluster for an uncharacterized chemotaxis two-component signaling system (Wadhams and Armitage, 2004), which is well conserved in the other *Thermosynechococcus* genomes.

### Other notable genetic features

Another notable genetic feature shared by TA-1 and CL-1 is apparent expansions of Uma2 (DUF820) family endonuclease and recombination promoting nuclease/putative transposase genes. The Uma2 domain-containing genes encode a family of putative restrictive endonucleases (Uma2 family endonucleases) (Kinch et al., 2005). TA-1, CL-1, and PKUAC-SCTE542 all have high copy numbers (16, 17, and 15, respectively) of Uma2 family endonuclease genes, whereas the three Japanese strains all have only four or fewer copies (Figure 3). In addition, the RPN family genes that encode recombination-promoting nuclease/putative transposases (Kingston et al., 2017) are greatly expanded in the two Taiwanese strains (Figure 3). RPN family genes in the TA-1 genome are located in three tandem gene arrays (containing 11, 3, and 3 copies, respectively). In comparison, the outgroup *S. lividus* has only one copy of the RPN family gene. Furthermore, RPN family genes are absent in genomes of JA-3-3-Ab and JA-2-3Ba (*Synechococcus* phylogenetic A/B lineage) from Yellowstone National Park (Bhaya et al., 2007). Taken together, these findings indicate the expansion of the *uma2* gene family in the MRCA of the Chinese/Taiwanese lineages and further expansion of the *rpn* gene family in the MRCA of the two Taiwanese strains.

## Conclusion

In this work, we reported the complete genome sequence of a thermophilic cyanobacterium (i.e., strain TA-1) and conducted comparative analysis with other representatives within the genus *Thermosynechococcus*. Compared with a previous work that examined the genetic differentiation of *Thermosynechococcus* in the context of environmental factors and found no clear link (Prondzinsky et al., 2021), we found that the genetic differentiation of *Thermosynechococcus* are mostly congruent with phylogeny. Moreover, the two strains collected in Taiwan represent a novel species-level taxon in this genus, and this taxon has several distinct genetic features (e.g., antibiotic resistance, EPS biosynthesis, and motility). However, despite the high genetic relatedness between these two strains, we observed extensive genome rearrangements and multiple differentiation of gene content, which indicates the dynamic nature of the genome evolution of these bacteria. Taken together, these findings improve our understanding of these cyanobacteria, which are the primary producers in non-acidic hot springs, and provide genomic resources for future functional characterization that can benefit biotechnology applications.

## Data availability statement

The datasets presented in this study can be found in online repositories. The names of the repository/repositories and accession number(s) can be found below: https://www.ncbi.nlm.nih.gov/genbank/, CP070960 and https://www.ncbi.nlm.nih.gov/, PRJNA702872.

## Author contributions

C-HK and H-AC: conceptualization, funding acquisition, project administration, and supervision. Y-IC and Y-CL: investigation, validation, and visualization. J-YL: biological materials. Y-IC, Y-CL, C-HK, and H-AC: writing – original draft. Y-IC, Y-CL, J-YL, C-HK, and H-AC: writing—review and editing. All authors contributed to the article and approved the submitted version.

## Supplementary material

Supplementary Figure 1 Morphology of *Thermosynechococcus* sp. TA-1 cells. **(A)** Light microscopy. **(B)** Scanning electron microscopy. **(C)** Transmission electron microscopy, longitudinal section. **(D)** Transmission electron microscopy, partial enlargement of the longitudinal section. **(E)** Transmission electron microscopy, transverse section.

Supplementary Figure 2 Growth of *Thermosynechococcus* sp. CL-1 and TA-1 in BG-11 medium with or without bicarbonate. Error bars are plotted but not discernible at this scale.

Supplementary Figure 3 Growth of *Thermosynechococcus* sp. TA-1 in BG-11 medium with nitrate or nitrite as the nitrogen source. Error bars are plotted but not discernible at this scale.

Supplementary Figure 4 Growth of *Thermosynechococcus* sp. **(A)** TA-1 and **(B)** CL-1 on solid agar plates containing 5 μg/ml of kanamycin under directional white light.
