# Supplementary figures and images for: Comparative analysis reveals distinctive genomic features of Taiwan hot-spring cyanobacterium Thermosynechococcus sp. TA-1
Source: Front Microbiol. 2022 Aug 11;13:932840. doi: 10.3389/fmicb.2022.932840 (PMC9403480; doi:10.3389/fmicb.2022.932840)

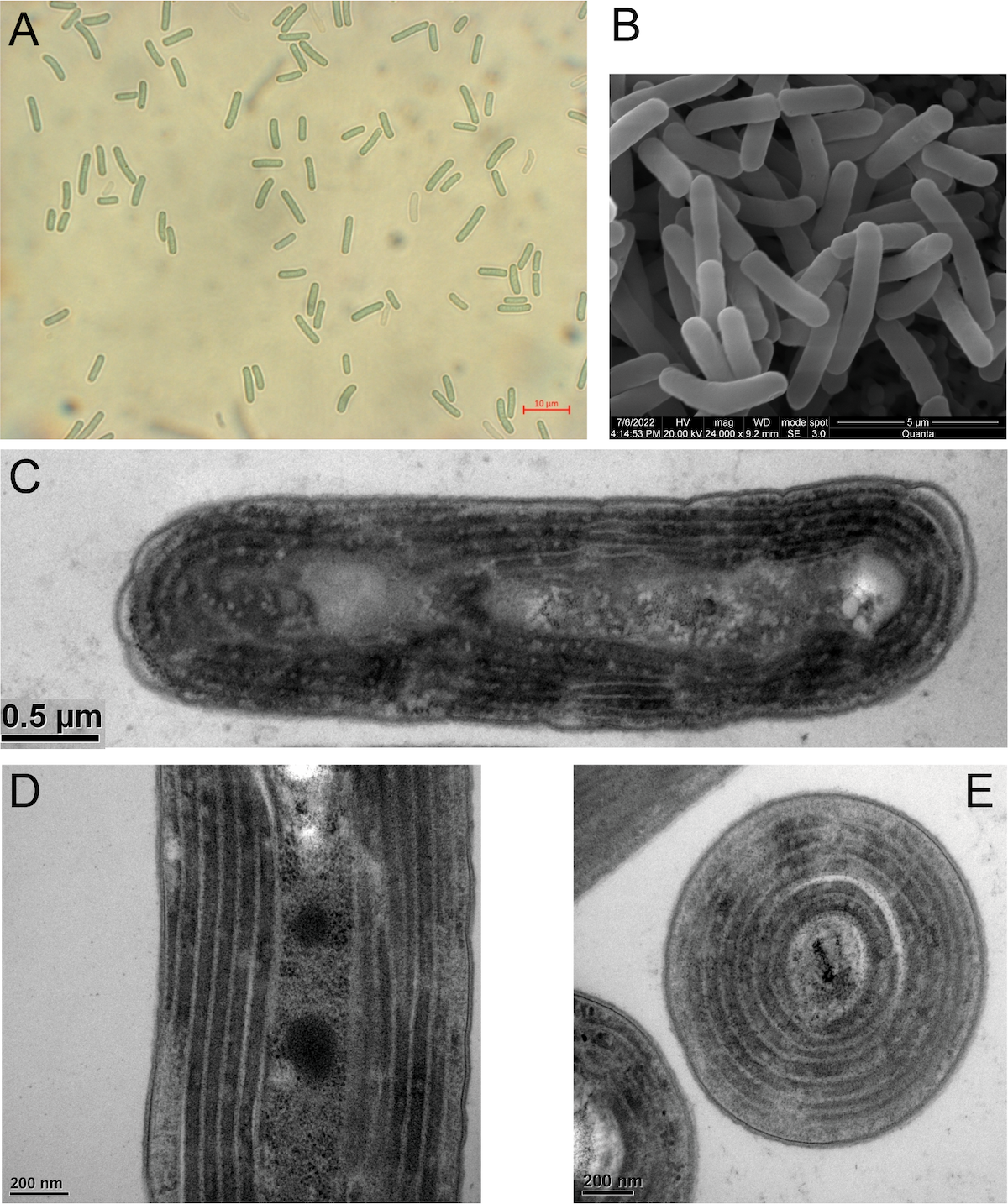

Supplement: Supplementary Figure 1 — Morphology of Thermosynechococcus sp. TA-1 cells. (A) Light microscopy. (B) Scanning electron microscopy. (C) Transmission electron microscopy, longitudinal section. (D) Transmission electron microscopy, partial enlargement of the longitudinal section. (E) Transmission electron microscopy, transverse section. [file Image_1.TIF]

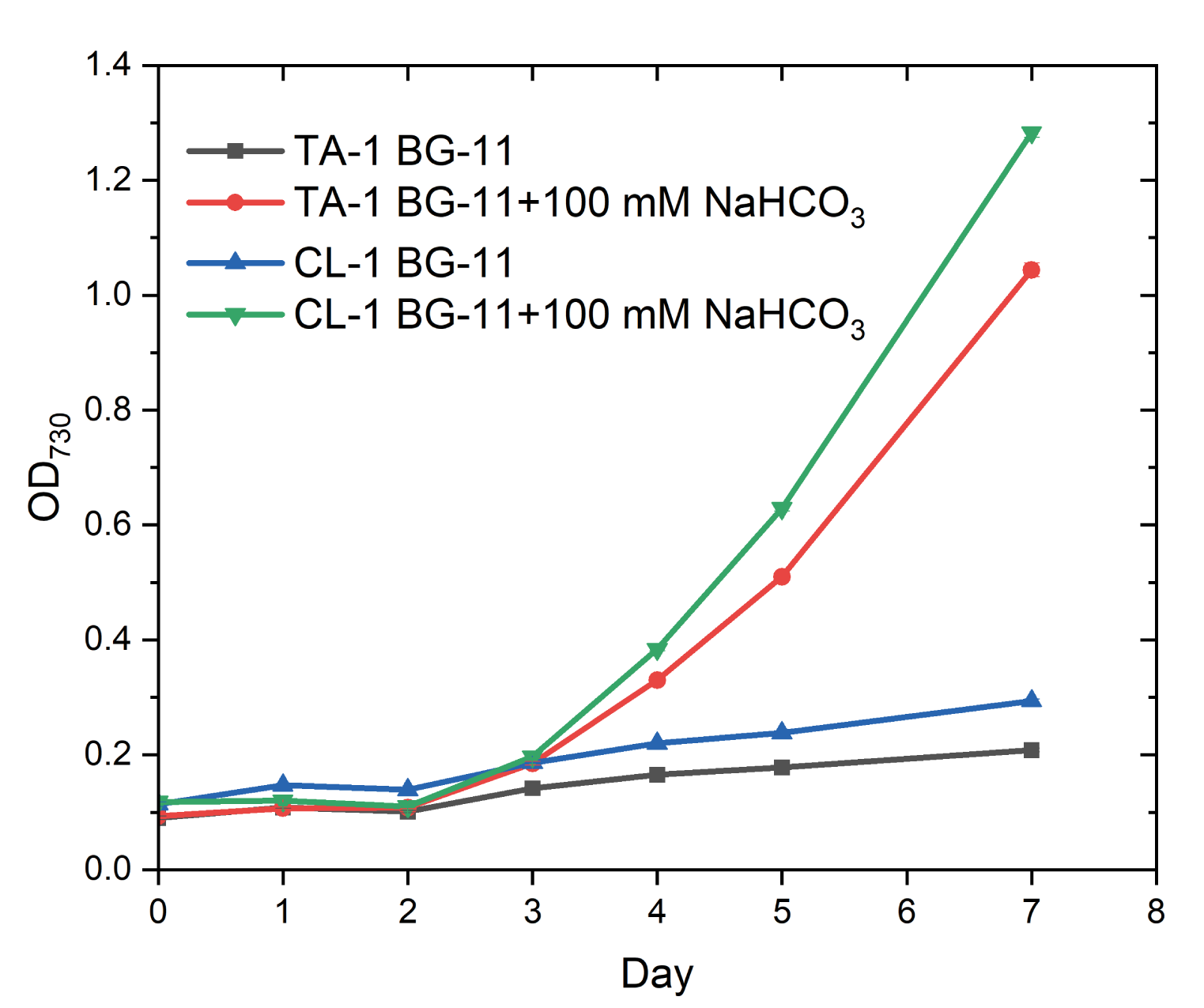

Supplement: Supplementary Figure 2 — Growth of Thermosynechococcus sp. CL-1 and TA-1 in BG-11 medium with or without bicarbonate. Error bars are plotted but not discernible at this scale. [file Image_2.TIF]

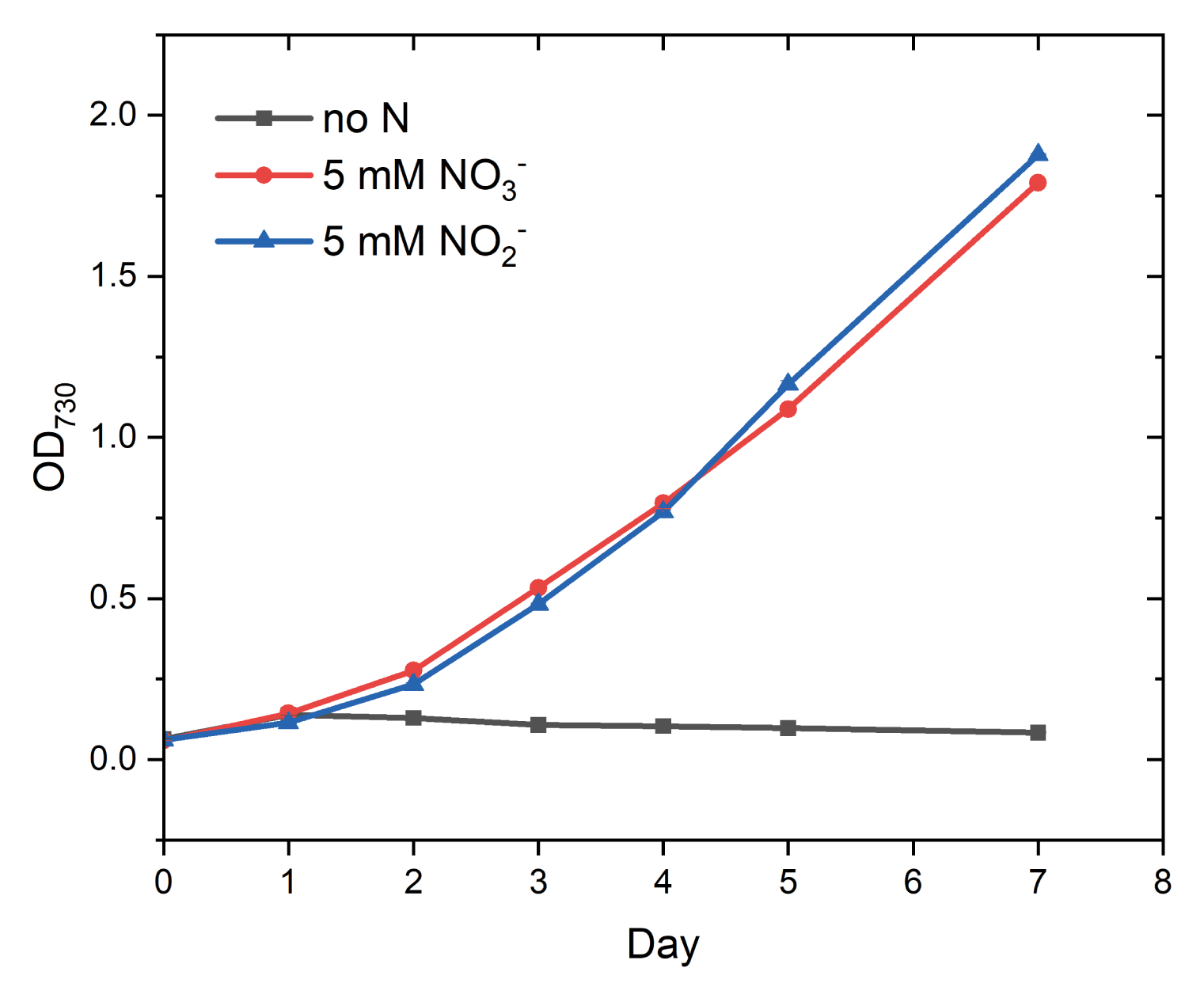

Supplement: Supplementary Figure 3 — Growth of Thermosynechococcus sp. TA-1 in BG-11 medium with nitrate or nitrite as the nitrogen source. Error bars are plotted but not discernible at this scale. [file Image_3.TIF]

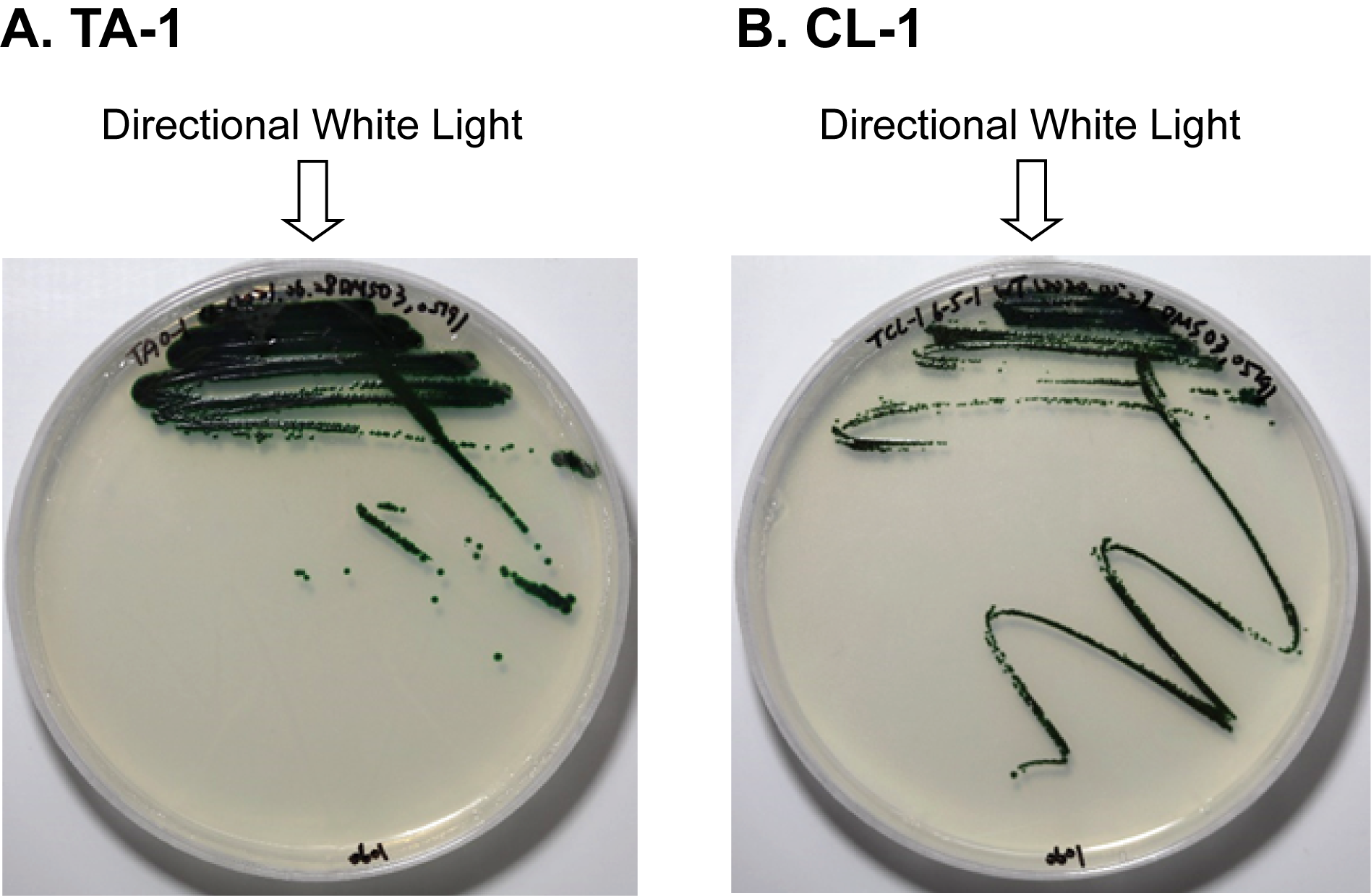

Supplement: Supplementary Figure 4 — Growth of Thermosynechococcus sp. (A) TA-1 and (B) CL-1 on solid agar plates containing 5 μg/ml of kanamycin under directional white light. [file Image_4.TIF]
